# Population connectivity, dispersal, and swimming behavior in *Daphnia*

**DOI:** 10.1002/ece3.7246

**Published:** 2021-02-28

**Authors:** Sylvie V. M. Tesson, Yongcui Sha

**Affiliations:** ^1^ Department of Biology Aarhus University Aarhus Denmark; ^2^ Department of Biology Lund University Lund Sweden

**Keywords:** behavioral plasticity, landscape barriers, local adaptation, microsatellites, population genetics, refuge demand

## Abstract

The water flea *Daphnia* has the capacity to respond rapidly to environmental stressors, to disperse over large geographical scales, and to preserve its genetic material by forming egg banks in the sediment. Spatial and temporal distributions of *D*. *magna* have been extensively studied over the last decades using behavioral or genetic tools, although the correlation between the two has rarely been the focus. In the present study, we therefore investigated the population genetic structure and behavioral response to a lethal threat, ultraviolet radiation (UVR), among individuals from two different water bodies. Our results show two genetic populations with moderate gene flow, highly correlated with geographical location and with inheritable traits through generations. However, despite the strong genetic differences between populations, we show homogeneous refuge demand between populations when exposed to the lethal threat solar UVR.

## Graphical abstract

The study shows two genetic populations with moderate gene flow, highly correlated with geographical location and with inheritable traits through generations. Despite the strong genetic differences between populations, we show homogeneous refuge demand between populations when exposed to the lethal treat solar UVR.

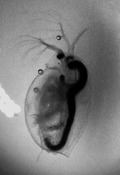

## 1 INTRODUCTION

A paradox of the zooplankter *Daphnia* is its strong degree of provincialism (De Gelas & De Meester, 2005) and behavioral patterning (Ekvall et al., 2015) occurring simultaneously to its high capacity of moving (e.g., vertical diurnal migration) and dispersal (Hansson et al., 2016; Pinceel et al., 2016; Waterkeyn et al., 2010), as well as its ability to adapt to changing environments (e.g., plasticity (Kim et al., 2009; Miner & Kerr, 2011)) and revival (Hairston et al., 1995).

*Daphnia* has a high potential of movement and dispersal. Propagules can be transported by flowing water (Havel & Shurin, 2004), air (Caceres & Soluk, 2002; Pinceel et al., 2016; Vanschoenwinkel et al., 2008), and animals (van de Meutter et al., 2008; Waterkeyn et al., 2010). They can rapidly colonize new environments even at local scales (Louette & De Meester, 2004, 2005; Michels et al., 2001). However, the distribution of species or populations is restricted over geographical scales by environmental selection and priority effects affecting genes under selection, gene flow among populations, and the genetic diversity within populations (Orsini et al., 2013). Moreover, isolation by distance occurs along geographical scales when spatial restrictions are coupled with long‐term dynamic of distance‐dependent dispersal (Fields et al., 2015).

Locally, *Daphnia* can respond rapidly to threats. Predation and environmental factors, such as ultraviolet radiation (UVR), act in concert to shape its swimming behavior resulting in avoidance migrations (Ekvall et al., 2020; Hansson & Hylander, 2009a; Lampert, 1989; Williamson et al., 2011; Williamson et al., 2001). Such escape behavior affects the vertical pattern through the water column (Ekvall et al., 2015; Hansson & Hylander, 2009b), impacting its refuge demand (Hansson et al., 2016) and the ecology of the ecosystem (e.g., Grossart et al., 2010). Moreover, *Daphnia* can adapt to multiple environmental stressors by adjusting its morphology, swimming behavior, and life history traits within a few parthenogenic generations (Sha et al., 2020). In addition to phenotypic plasticity (Christjani et al., 2016), recent studies have suggested the occurrence of personalities among *Daphnia* individuals when exposed to UVR (Heuschele et al., 2017).

The concept of personality has been proposed to explain individual variance within ecological populations, for example, with respect to the strength of behavioral responses (Chapman et al., 2011). However, although the fields of behavioral response and personalities have retained much attention lately, the genetic basis of behavioral responses is rarely addressed. Plaistow and Collin (2014) stressed the importance of investigating multivariate phenotypic plasticity (behavior, physiology, and morphology) associated with the genetic differences within clonal lineages. To our knowledge, only one study has investigated the genetic fingerprint associated with swimming behavior in response to stressors, showing an increased plasticity in accordance with adaptive evolution (Cousyn et al., 2001). Hence, there is a lack of understanding with respect to the genetic behavioral fingerprint in *Daphnia* and its sustainability within natural populations.

In this study, we assessed the behavioral responses in *Daphnia magna* associated with natural diel vertical migration in relation to their genetic fingerprints at the population and individual levels. Because lakes in the Scania region have different history and are influenced by anthropogenic activity (Janson et al., 2014), we expected a presence of multiple genetic populations of *D. magna* in the investigated water systems, with little correlation over geographical location. In each water body, we also expected meta‐population structure characterized by different genetic clusters with specific behavioral signatures associated with local adaptation (e.g., nutritional requirements (Maruki et al., 2019)). Moreover, we hypothesized that individuals belonging to the same population genetic cluster would exhibit similar swimming behavior and life traits, inheritable across generations.

## 2 MATERIALS AND METHODS

### 2.1 Organismal collection and cultivation

**FIGURE 1:**
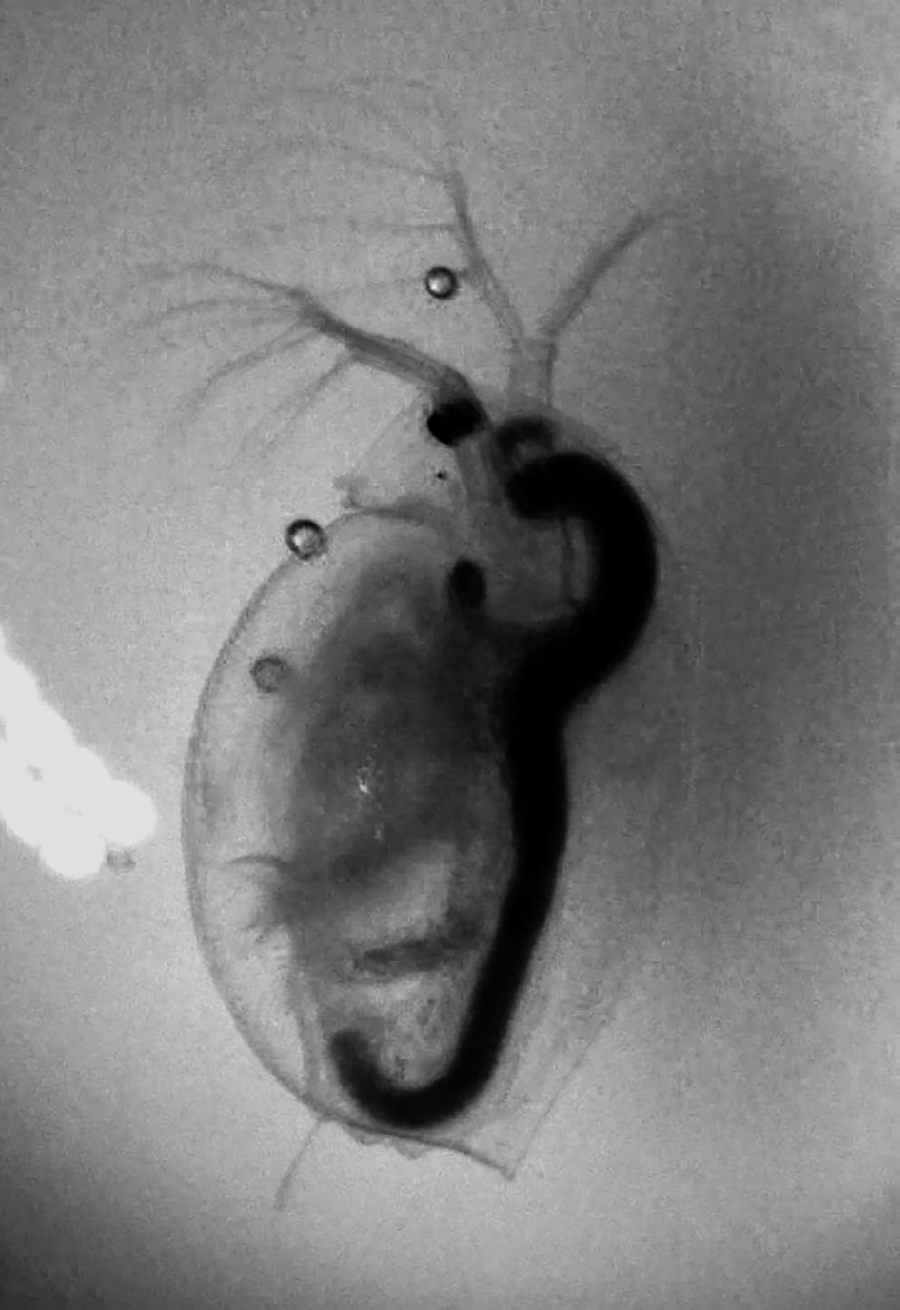
Female *Daphnia magna* observed for sex and taxon determination using a stereomicroscope

**FIGURE 2:**
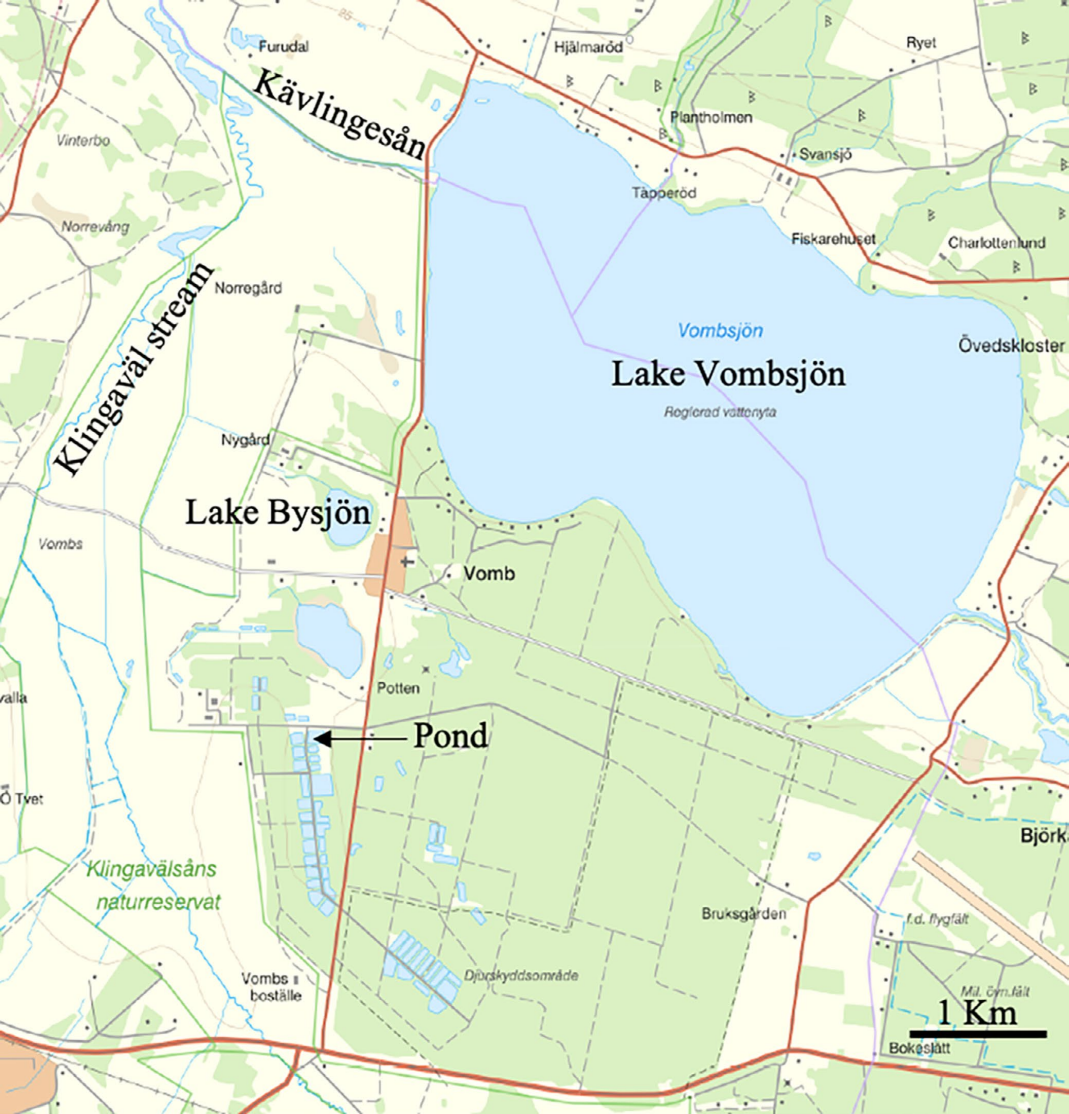
Sampling location at Lake Bysjön (harboring mainly POP1 individuals) and Sydvatten Pond (harboring mainly POP2 individuals), Scania region, southern Sweden

*Daphnia magna* (Figure 1) were collected in Lake Bysjön (55.67424 N–13.546529 E) and in a Sydvatten pond (55.660709 N – 13.541140 E), located less than 3 km apart from each other, in southern Sweden (Figure 2). Lake Bysjön is a hypertrophic seepage lake alimented by groundwater inflow (Enell, 1982; Persson & Svensson, 2004), whereas the human‐made Sydvatten pond is part of a water network, involved in the production of drinking water for consumption. It receives filtered water (mesh 40 µm) from the nearby Lake Vombsjön (Sydvatten communication). Environmental parameters and information on the presence of fish are given in Table 1. Organisms were sampled using a 200‐µm meshed plankton net and individually isolated under a stereomicroscope (SZX7, Olympus). Gender identification and taxonomy were determined using Infinity Analyse and Capture software (version 6.5.4) and reference taxonomic keys (Chiang & Du, 1979; Lilljeborg, 1901). A total of 35 individuals of *D. magna* were collected (Appendix S1) and grown at 18°C under a 14:10‐hr light:dark cycle of 50 µmol photon m^−2^ s^−1^ in a 100‐ml glass bottle (Heraco AB 6,212, Holmsund, Sweden) filled with 60 ml copper‐free water and were fed every 2–3 days with 10^5^ cells of pure culture of *Scenedesmus* sp. At each reproduction cycle (clutch), the mother was transferred to a new culture bottle and the offspring were grown for a week prior to being transferred to separate culture bottles, thereby constituting the next generation. Only females were kept for the analyses.

**TABLE 1:** Environmental parameters collected at each sampling locations. The location name (Location), number of strains of *Daphnia magna* collected (*N*_s_), the pH, conductivity, temperature, depth at the location, period of sampling (Period), and presence of possible fish predators

Location	*N*_s_	pH	Conductivity (µS/cm)	Temperature (°C)	Depth (cm)	Period	Presence of fish
Lake Bysjön	11	8.8–9.1	316–352	13.3–14	119	May 2015	Yes
Pond Sydvatten	24	9.1–9.6	281	14.1	50	May 2017	No

### 2.2 Swimming behavior

In order to account for maternal effects, clonal lineages were grown for three consecutive parthenogenic generations prior to behavioral analyses (Lynch, 1985). Individual swimming behavior was monitored in a Plexiglas aquarium (0.2 × 0.2 × 0.85 m) filled with 30 L of copper‐free water resulting in a water column of 0.75 m (Palmér et al., 2016) illuminated from above by LED‐470 nm at 50 µmol photons m^‐2^ s^‐1^ to mimic nighttime and with addition of 150 mA UVR (UVA‐340, Q‐PANEL Co, USA) to mimic solar radiation during daytime (Ekvall et al., 2013). Individuals were labeled using fluorescent Quantum dots (585 ITK Carboxyl Quantum dot, Life Technologies, Prod. No. Q21311MP) and then transferred to the monitoring aquarium, where their swimming behavior was recorded under three consecutive illumination phases, including one minute of LED illumination (Acclimation phase), followed by one minute of UVR (Stimulation phase) and by a one minute of LED illumination (Recovery phase) (Sha et al., 2020). From these video recordings, we extracted the 3D position of each individual at six frame per second in MATLAB V1.7 using the method described in Palmér et al. (2016). We then calculated the swimming speed and the refuge demand (mm × s), that is, cumulative vertical position through the three‐illumination phases (Hansson et al., 2016). We calculated the individual mean values of swimming speed for each UVR phase for later statistical analyses. Dataset includes results from swimming behavior and refuge demand across generations for Lake Bysjön and at the third generation in the Pond (Appendix S1). Statistical comparisons between genetic populations and lineages were performed in R v4.0.2 (R Core Team, 2020) using analysis of variance (ANOVA) on normal and homogeneous variance of residuals, and nonparametric tests for unbalanced design: the Kruskal–Wallis rank sum test (one‐way analysis of variance), the Student two‐samples *t* test for equal variances, and Welch test for unequal variances.

### 2.3 Extraction of genetic material

After tracking, organisms were individually frozen at −20°C. Their genomic DNA was extracted following a modified protocol from Richlen and Barber (2005). Each individual was placed in 100 µl solution of sterile Chelex 10% (Chelex 100 Bio‐Rad, diluted in double distilled water and autoclaved 121°C 30 min). Samples were vortexed for 5 s, centrifuged for 20 s (3,300 *g*), incubated at 95°C for 20 min, vortexed for 5 s, and spun down. The supernatant was collected for genetic analyses. Extracted DNA was quantified using dsDNA HS Qubit Assay Kit (Q32854, Life Technologies Corp., Eugene, Oregon, USA).

### 2.4 Libraries and database preparation

Genetic amplifications using polymerase chain reaction (PCR) were performed in 12.5 µl final volume using a modified protocol from Colson et al. (2009) with a final concentration of 1.4× PCR buffer, 2.5 mM MgCl_2_, and 0.08 to 8 ng of DNA. Twenty‐six microsatellites markers were amplified using four multiplexes developed by Orsini et al. (2012) (Table 2). PCR conditions of multiplexes M01, M05, and M06 comprised a denaturation step at 94°C for 4min, 30 cycles of denaturation (94°C for 30 s), annealing (56°C for 30 s) and extension (72°C for 30 s), and a final extension at 72°C for 4 min. For multiplex ESTM01, 35 cycles were used with an annealing temperature of 54°C. After amplification, samples were vortexed shortly and spun down, and a volume of 6 µl of amplicons was sent for sequencing to the Uppsala Genome Center, Sweden. Microsatellite fragments were analyzed using Geneious Prime 2020.0.3 software (https://www.geneious.com). GeneScan™ 600 LIZ™ dye Size Standard v2.0 (Thermo Fisher) was used as reference. Noise was removed from microsatellite dataset using a three‐step filtration pipeline by removing: (a) microsatellite markers with >30% of null alleles (i.e., 10 loci), (b) individuals with >30% of null alleles (40 individuals), and (c) monomorphic loci (1 locus). The final dataset was composed of haplotypes of 137 individuals (Lake Bysjön: 11 and Pond: 24 strains) over 15 microsatellite markers (Appendix S2).

**TABLE 2:** Information on microsatellite markers. Genetic marker, chrome, core motif, expected fragment length from previous studies, multiplex reference, and literature reference (source)

Marker	Chrome	Core	Fragment length	Multiplexes	Source
**A002**	6FAM	(AC)9	250–282^a^; 262–274^b^	M05	N^b^, ^c^
**A009**	NED	(GAA)6	216–280^a^; 231–279^b^	M06	N^b^, ^c^
**B008**	VIC	(TC)9	148–174^a^; 150–170^b^	M01	N^b^
**B030**	PET	(GA)11	150–174^a^; 154–172^b^	M01	N^b^
**B033**	NED	(TG)8	85–129^a^; 96–114^b^	M05	N^b^, ^c^
B045	NED	(TG)8	110–130^a^; 118–126^b^	M01	N^b^
**B050**	6FAM	(GAA)6	220–250^a^; 234–248^b^	M01	N^b^
B052	PET	(CA)13	181–215^a^; 277–305^b^	M05	G^b^, ^c^
**B064**	6FAM	(TC)8	130–160^a^ ; 135–151^b^	M01	N^b^
**B074**	NED	(GT)9	180–210^a^ ; 196–204^b^	M01	N^b^
**B081**	PET	(TAA)4	150–190^a^ ; 188–200^b^	M06	N^b^, ^c^
**B087**	6FAM	(CA)13	174–200^a^ ; 184–192^b^	M05	G^b^
**B096**	VIC	(AC)15	225–245^a^ ; 234–240^b^	M01	N^b^
**B097**	6FAM	(AC)15	241–283^a^ ; 264–272^b^	M06	F^b^, ^c^
B107	PET	(CT)8	242–290^a^ ; 250–274^b^	M01	G^b^, ^c^
B168	VIC	(CT)5	170–216^a^; 403–409^b^	M06	N^b^, ^c^
B179	VIC	(CA)9	380–420^a^ ; 339–361^b^	M06	G^b^, ^c^
**B180**	VIC	(ATTT)4	264–308^a^ ; 301–311^b^	M05	N^b^, ^c^
WFes0001508	PET	(AG)10‐5	127–171^a^	ESTM01	G^d^
WFes0002936	6FAM	(TTC)6‐5	250–280^a^	ESTM01	N^d^
WFes0004276	NED	(ATT)18‐11	260–320^a^	ESTM01	N^d^
**WFes0005005**	NED	(TG)10‐10	100–140^a^	ESTM01	N^d^
WFes0006166	VIC	(CA)12‐9/(TAT)6‐6	156–180^a^	ESTM01	N^d^
**WFes0008344**	6FAM	(CT)8‐8	100–140^a^	ESTM01	N^d^
WFes0008608	PET	(CAA)16‐5	276–310^a^	ESTM01	^d^
WFes0009235	VIC	(TGG)5‐5	290–330^a^	ESTM01	^d^

Note

The 15 markers retained for the analyses are indicated in bold. Markers identified in Orsini et al., 2012 as neutral (N) or associated with fish predation (F), parasite infection, and land use stressors (G).

^a^ Orsini et al. (2012).

^b^ Jansen et al. (2011).

^c^ Routtu et al. (2010).

^d^ Colson et al. (2009).

### 2.5 Population genetic analyses

Screening for scoring errors (large dropout, null alleles, stuttering) and allele distribution were made using Micro‐Checker software version 2.2.3 (Van Oosterhout et al., 2003) with 95% confidence intervals, a Bonferroni correction (Dunn–Sidak), and 1,000 permutations (Appendix S3). We successively used the set of 13 neutral markers (Table 2) to investigate population genetic structure and connectivity processes and the two adaptive markers (Table 2), under influence of fish predation stress (Locus B097) and general landscape stressors (fish predation, parasite infection, and land use stressors; Locus B087), to identify potential correlations with organismal swimming behavior in inferred populations.

Canonical factorial correspondence analysis was performed using the Genetix software, version 4.05.2 (Belkhir et al., 1994). The number of genotypes was inferred using the Gimlet software version 1.3.3 (Valière, 2002), the function "regroup genotypes," and the option "missing alleles as any other alleles." The number of populations and their genetic structure were investigated using the Structure version 2.3.4 (Pritchard et al., 2000) on strictly neutral markers with a burn‐in period of 20,000 iterations, a MCMC after burn‐in of 20,000 iterations, admixture model, a number of 20 replicates, and no prior information on location or ancestry. The number of populations was inferred using the Evanno method (Evanno et al., 2005).

Genetic diversity within and between populations was investigated using both Genetix and GenAlEx software version 6.51b2 (Smouse et al., 2017). Allele richness, heterozygosity, *F*‐statistics, the number of private alleles, and fixation index across loci were calculated for codominant markers. The inbreeding coefficient was calculated within populations (*F*_ST_) and individuals (*F*_IS_), for small population size (*G*_‐statistics_), and with a stepwise mutation model approach (*R*_ST_), with 1,000 permutations. The number of effective migrants (*N_m_*) was estimated between populations using codominant data and Equation 1 (Wright, 1969). The Hardy–Weinberg equilibrium was tested in population with sample size ≥50 and expected numbers per classes ≥5. The degree of allelic association between locus was assessed by measuring linkage disequilibrium (1,000 bootstrap).

(1) $$N_{m}=\frac{1‐F_{\text{ST}}}{4F_{\text{ST}}}$$

Bottleneck effect in inferred populations was investigated using the BOTTLENECK software version 1.2.02 (Cornuet & Luikart, 1997) and the neutral markers. The *two‐phase model* (TPM) for multiple‐step mutations was applied to the data with default settings recommended for microsatellites analyses (i.e., a variance of geometric distribution of 30 and a proportion of stepwise mutations of 70%) and 1,000 permutations. A two‐tailed Wilcoxon sign rank test (Luikart et al., 1998) was applied to each population to detect heterozygosity excess and recent bottlenecks.

## 3 RESULTS

In the two water systems, 35 strains (Lake Bysjön: 11 strains; Pond: 24 strains), represented by 137 individuals, were genetically characterized over 15 polymorphic microsatellite loci, including 13 neutral and two adaptive markers (Table 2). Each locus was represented by a set of 2 to 4 alleles (Appendix S3).

The genotypic proportions of all neutral loci in each location were in the Hardy–Weinberg equilibrium (*F*_IS_B_ = −0.0008, *F*_IS_P_ = −0.0537) with little influence of inbreeding avoidance. There was a moderate gene flow between geographical locations (*F*_st_ = 0.12 and a Raufaste and Bonhomme correction (RH’) of 0.13) with few expected migrants (*N_m_* = 1.77). The number of individuals sampled per locus was variable in the two locations (*N*_Lake Bysjön_ = 46 ± 3; *N*_Pond_ = 76 ± 7), but in sufficient number to perform a Wilcoxon sign rank test. A significant heterozygote excess was detected in the Pond (*p* < 0.001, Wilcoxon sign rank test with 1,000 permutations; Table 3). The distribution of allele frequency followed a normal L‐shape in Lake Bysjön, as expected under mutation–drift equilibrium, and a shifted mode in the Pond, as a sign of recent bottleneck effect.

**TABLE 3:** Wilcoxon sign rank tests for heterozygosity excess in *Daphnia magna* from natural populations using strictly neutral microsatellite markers

Site	*N*	*S*	*L*	*N*_s_	*N*_a_	*H*_e_	Wilcoxon sign rank test using TPM					
							LHe_obs_	LHe_exp_	*p*_exc_	*p*_def_	*p*_2tails_	Model
Lake Bysjön	51	11	13	46 ± 3	2 to 4; 3.2 ± 0.6	0.44 ± 0.16	9	7.31	0.19	0.83	0.38	NL
Pond	86	24	13	76 ± 7	2 to 4; 2.8 ± 0.8	0.49 ± 0.19	12	6.59	**<0.001**	0.99	**<0.001**	Sh

Note

Sampling location (Site); number of individuals (*N*) and strains (*S*); number of polymorphic loci (*L*); range and mean number of individuals sampled per locus (*N*_s_); allele per locus (*N*_a_); Hardy–Weinberg expected heterozygosity (*H*_e_); two‐phase model (TPM); number of loci with observed (LHe_obs_) and expected (LHe_exp_) heterozygosity excess; probability for heterozygosity excess (*p*_exc_), deficiency (*p*_def_), and both (p_2tails_); allelic frequency distribution model using mode‐shift indicator (Model) resulting in either a shifted (Sh) or normal L‐shaped (NL) distribution. Significant values of probability (alpha 5%) are in bold.

**FIGURE 3:**
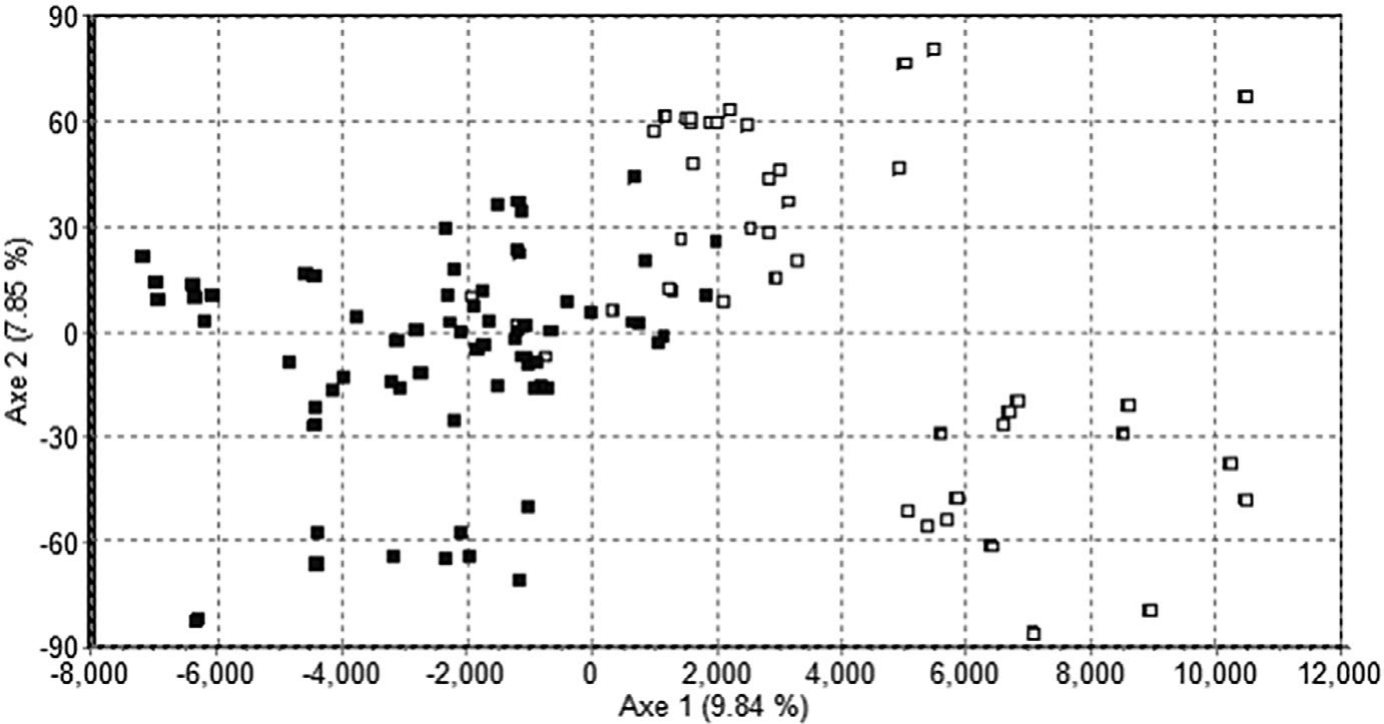
Canonical factorial correspondence analysis of 137 individuals from Lake Bysjön (51 individuals, white squares) and Pond (86 individuals, black squares)

Factorial correspondence analyses highlighted the existence of a couple of overlapping genetic clusters mostly following location prior (Figure 3). Population genetic structure analysis on the neutral markers and the 137 individuals without location prior confirmed this pattern and demonstrated the existence of two genetically distinct populations (POP1, POP2) virtually sorting the individuals based on sampling location. Indeed, almost all individuals with an origin from Lake Bysjön (90%) belonged to POP1 and from the Pond (97%) to POP2 (Appendix S1). The remaining four individuals shared less than 80% of genetic similarity to either of the two genetic clusters (66%–73%; Appendix S1) and were classified as population Hybrids. This clear separation of individuals into population according to sample location indicates a strong influence of geographical barriers, despite the close distance between the locations (Figure 2). On the other hand, the presence of Hybrids reflects some gene flow and individual dispersal between locations.

Population isolation by distance between POP1 and POP2 was inferred using pairwise differentiation indexes. There was a moderate genetic differentiation between POP1 and POP2 with a *F*_ST_ of 0.13 and a RH’ equal to 0.15. Furthermore, the estimated number of migrants between populations was 1.62 (Weir and Cockerham *F*_ST_ estimate) highlighting the existence of gene flow between populations.

Each genetic population was characterized by a pool of 37 (POP1) to 38 (POP2) alleles, a set of 2 to 4 alleles per locus (POP1: 2.9 ± 0.7 alleles, POP2: 2.9 ± 0.8 alleles), and few private alleles (POP1: 7 alleles; POP2: 8 alleles) (Appendix S3). The allele frequency distribution in POP1 showed a more attenuated L‐shape than in Lake Bysjön, and POP2, a clear mode shift distribution similar to the Pond. Nonetheless, the two populations were in the Hardy–Weinberg equilibrium (HWE), indicating that individuals were randomly mating with a slight apparent tendency for inbreeding avoidance (*F*_IS_POP1_ = −0.007, *F*_IS_POP2_ = −0.050, 1,000 permutations).

Correlation analysis between pair of loci showed signs of random association between alleles at different loci. Random associations (with *r* ^2^ > 5% threshold) were present in 45% (POP1) and 40% (POP2) of the locus pairwise comparisons with 1 to 8 random associations per locus in POP1 (5.4 ± 2.3) and 1 to 7 in POP2 (4.8 ± 1.9) (Appendix S3). Significant linkage disequilibrium (*p* < .05, Appendix S4) occurred in both populations over two loci (B008 and B030) and at four additional loci in POP1 (Loci: A009, B033, WFes0005005, and WFes0008344) and in POP2 (Loci: B074, B081, B096, and B180), respectively. There was no sign of stuttering or allele dropouts in the dataset (Appendix S3). However, the presence of null alleles may have contributed to the elevated linkage disequilibrium observed in POP1 at Locus WFes0005005 and in POP2 at Locus B030 and Locus B180.

Genotypic diversity was investigated over the 13 neutral markers in the 137 individuals. Neutrals markers do not undergo selection and therefore highlight genetic variation of a population (Holderegger et al., 2006). A total of 46 unique genotypes were identified. The majority of these genotypes belonged to one of the inferred genetic clusters, that is, 30% to POP1, 59% to POP2, and 4% to the Hybrid group (Appendix S1). Only three genotypes were shared between genetic clusters, that is, gr.9, gr.11, and gr.46, possibly due to the presence of null alleles (Appendix S5). Moreover, each genotype was strain‐specific (Appendix S1, Appendix S5), with the exception of gr.2 (B36‐P07).

**FIGURE 4:**
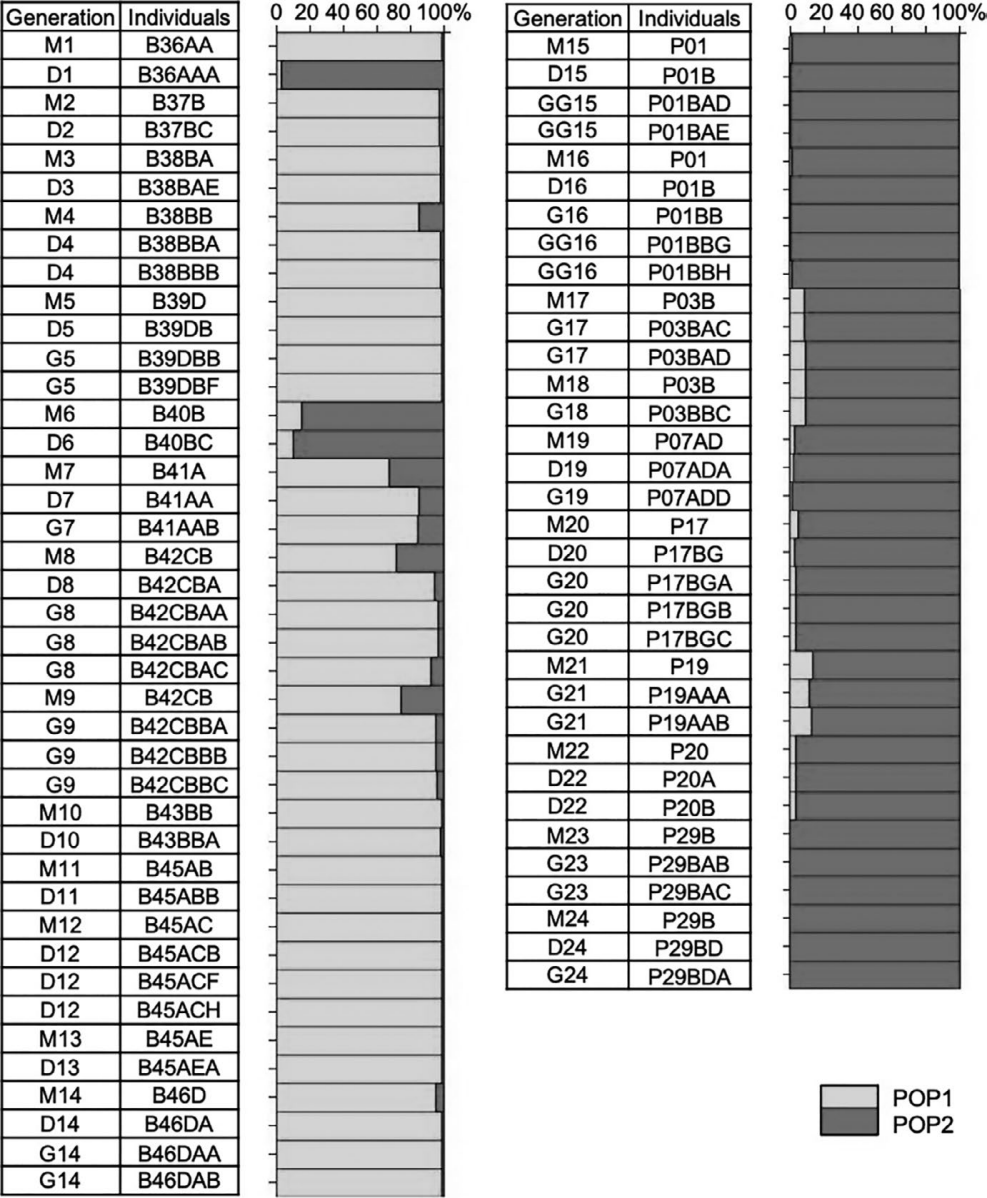
Distribution of population genetic diversity through 24 lineages based on neutral markers. Lineages are composed of mothers (M), daughters (D), granddaughters (G), and sporadically of great‐granddaughters (GG). Bars indicate the probability of an individual to belong to either POP1 (light gray), POP2 (dark gray), or being a Hybrid of the two populations (proba < 80%)

Using prior information on generation, we investigated the population genetic inheritance and mutation pattern in 24 clonal lineages, that is, mother–daughter–granddaughter–great‐granddaughter. In parthenogenetic reproduction, a high number of individuals are expected to transmit their genetic information intact to the next generation. Results showed that affiliation to population genetic clusters was maintained through generations in almost all investigated lineages (20 out of 24 lineages; Figure 4). Moreover, 75% of the investigated lineages met the expectation that descendants exhibited the same genotype as their mother up to four generations (Appendix S6). Deviation from the expected inheritance pattern can be due to sexual recombination. However, *Daphnia magna* is an obligate asexual reproducer and the study has been designed to remove any occurrence of sexual reproduction. Further deviation can be the result of the absence of gene amplification (null alleles) or the acquisition of genetic mutations in polymorphic microsatellite markers, which lead to an increase in heterozygosity in asexual lines (Seyfert et al., 2008). Null alleles occurred in the dataset and may be at the origin of the observed transition in population genetic clusters in strain P31 (Appendix S6). The removal of strain P31 from the dataset did not affect the population estimates and was therefore kept for further analyses. Another transition to population genetic clusters was observed in strain B36 influenced by the acquisition of mutation events over one generation and multiple loci. Globally, the locus‐based mutations in all mutated strains lead to a net gain of heterozygosity (Appendix S6). Expected microsatellite mutations are often characterized by one to several step mutations at one or both alleles at a given locus. Observed mutations were characterized either by an insertion/deletion of one allele (e.g., 7 core repeats in B42 lineage 8 at Locus A009), by one‐ up to three‐step mutations at one allele (80% of the cases), or by frameshift mutations on both alleles (e.g., P01 lineage 15 at Locus B008 and P07 at Locus A002) (Appendix S6). Likely these events occurred after the mitotic cell division in B42, P01, and P07 as only one of the investigated siblings carried the mutations (i.e., B42CBAB versus B42CBAA and B42CBAC, P01BAE versus P01BAD, and P07ADA versus P07ADD). Unexpectedly was the acquisition of multiple mutation events in lineage B36 characterized by step and frameshift mutations over up to eight neutral loci in only one generation (Appendix S6).

Adaptive markers are utilized to infer adaptative or evolutionary potential of populations (Holderegger et al., 2006). Linkage disequilibrium between the two adaptive markers in the dataset was only marginal (*p* = .07; Appendix S4). However, they showed significant linkage disequilibrium with five neutral loci (*p* < .01; A002, B008, B030, B096, and WFes0005005), and the general adaptive marker (Locus B087) with four additional neutral loci (*p* < .001; B033, B064, B974, and B081) (Appendix S4). A total of seven genotypes were estimated over the two adaptive markers and the 137 investigated strains (Appendix S1). Adaptive genotypic diversity was maintained in all individuals in 64% of the investigated strains. Deviation from this pattern occurred through the acquisition of mutations both on Locus B097 (75% of the mutations) and on Locus B087 (25% of the mutations). Moreover, certain genotypes were population‐specific such as genotype Ab to POP1 and genotypes Bd, Be, and Cc to POP2. The latter three genotypes were acquired through step mutations on Locus B097 (i.e., P07BBA, P14BEE) or frameshift mutations on both alleles at Locus B087 (i.e., P07ACA).

**FIGURE 5:**
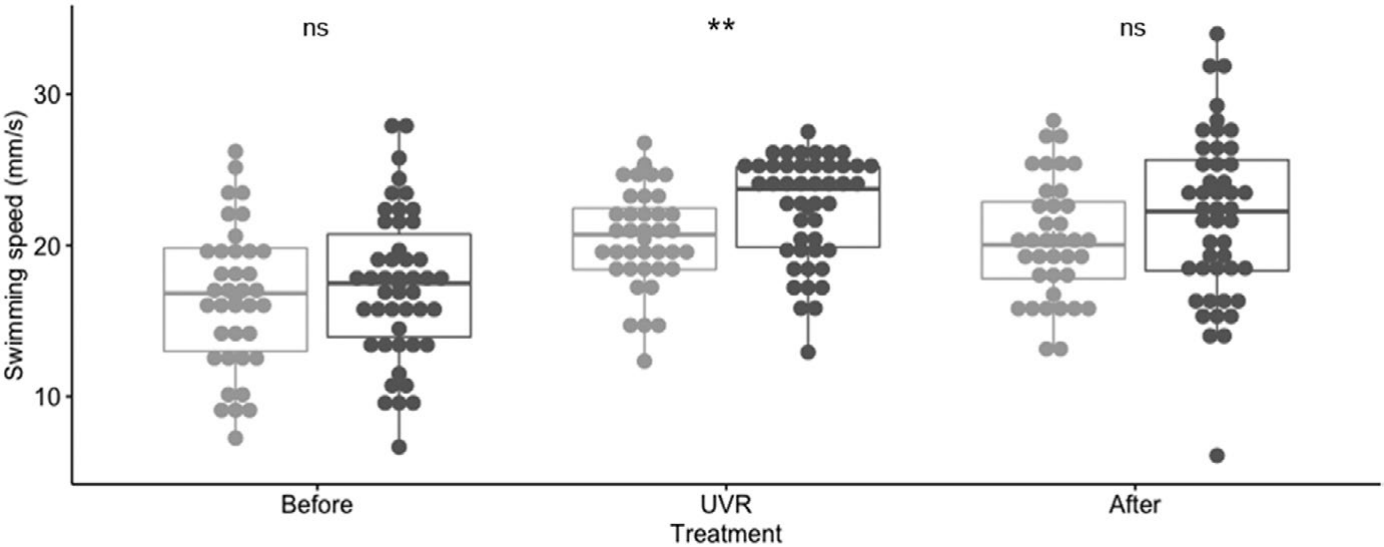
Swimming speed (mm/s) in individuals of *Daphnia magna* within genetic clusters (POP1 (light gray) and POP2 (dark gray)) over the three‐phase experiment in the presence (UV) and absence (before, after) of UV radiations. **indicates a significant difference in swimming speed (*p* < 0.01) between populations under threat, whereas ns stands for nonsignificant difference

The behavioral responses to UVR threat was investigated among individuals from the two genetic populations (POP1 and POP2). When exposed to UVR, there was a significant positive increase in swimming speed in both populations (Welch's two‐sample *t* test *t*_POP1_(64) = −4.07, *p*_POP1_ = 0.0001 and *t*_POP2_(81) = −5.73, *p*_POP2_ <0.0001) allowing individuals to flee away from the UVR source. Moreover, there was a significant difference in individual swimming speed between populations under UVR threat (Student's two‐sample *t* test, *t*_(81)_ = −2.73, *p* = 0.008; Figure 5). However, there was no significant difference in refuge demand between the two populations (Student's two‐sample *t* test, *t*_(81)_ = −0.43 *p* = 0.67). There was also no significant difference in refuge demand nor in swimming speed under UVR threat in either POP1 or POP2 across generation (Lake Bysjön; ANOVA *F*(2,26) = 0.18 *p* = 0.84, *F*(2,26) = 0.19, *p* = 0.83, respectively).

Additionally, organismal refuge demand and swimming behavior during the Stimulation phase were examined over the adaptive genotypes. There was no significant difference in refuge demand nor in swimming speed between the genotypes A and B (general stressor; Student's *t* test, *t*(76)_A‐B_ = 0.33 *p* = 0.74 and *t*(27)_A‐B_ = −1.51 *p* = 0.14, respectively). Similarly, there was no significant difference in swimming speed over the genotypes Ab, Ba, Bb, Bc, and Bd (Kruskal–Wallis rank sum test, *X* ^2^(4) = 1.31, *p* = 0.86). However, significant difference in refuge demand was observed between the genotypes Ba and Bd (ANOVA, *F*(4,56) = 2.80 *p* = 0.034, Tukey's HSD, *p* < 0.05).

## 4 DISCUSSION

High population diversity with consequent admixture events was expected because of the vicinity of the two water systems (less than 3 km apart) and of the intensive commercial and recreational use of aquatic environments in the region of Scania, southern Sweden. Such patterns have previously been confirmed in, for example, fish (crucian carp; *Carassius carassius*) (Janson et al., 2014). However, with respect to *Daphnia magna* our results show two clearly separated genetic populations almost perfectly matching with geographical locations (94% of individuals).

Despite being less than 3 km apart, the two water systems are isolated from each other by landscape barriers (Figure 2). Using seed‐banks of *D. magna*, a population genetic analysis demonstrated the existence of discrete genetic populations in isolated ponds, at different geographical scales (Orsini et al., 2013). Similarly, the absence of surface water connectivity between the two investigated water systems would explain why Lake Bysjön is mainly represented by POP1 individuals and the Sydvatten pond by POP2 individuals.

Isolation by distance promotes genetic differentiation between populations despite geographical barriers (Fields et al., 2015). Therefore, knowing the high dispersal rate of propagules, one would expect a higher gene flow in closely connected (Fields et al., 2015) or interconnected ponds (Orsini et al., 2013). At local scale, genetic distance analysis suggests that the two isolated but neighboring water systems were connected by a non‐negligible number of migrants and a moderate gene flow. It implies that Lake Bysjön and the Sydvatten pond are, with respect to *Daphnia* dispersal, in contact despite the absence of water connectivity.

Potential drivers for alternative ways of dispersal include transportation by wind, animals, and humans (Incagnone et al., 2014; Tesson et al., 2016) between different aquatic systems. Passive dispersal of zooplankters by natural vectors such as wind has been reported in previous studies (Havel & Shurin, 2004). The resistance of egg propagules (ephippia) to desiccation and freezing (Hebert, 1981) confers *Daphnia* with the ability to travel short distances (<5 m) up to several kilometers and to rapidly and intensively colonize isolated pools (Louette & De Meester, 2004, 2005; Sirianni, 2016).

Ephippia can also be transported passively by animals such as aquatic insects (van de Meutter et al., 2008), fish (Mellors, 1975), and birds (Figuerola et al., 2003). A phylogenetic analysis showed the importance of bird‐mediated dispersal and climate change in Arctic *Daphnia* species (Alfsnes et al., 2016; Haileselasie et al., 2016). The influence of such dispersal events on the genetic structure of zooplankton is rarely quantified, but at broader scale, our data suggest that although the effect is moderate since the two populations are still highly distinct, it may in the long run lead to both mixing of populations and hybridization.

Human can indirectly contribute to the dispersal of ephippia‐mediated fish stocking and the use of construction materials (Havel & Shurin, 2004). Resting stages of *Daphnia* can resist passage through the digestive apparatus of fish (Mellors, 1975) and be dispersed by fish stocking (Havel & Shurin, 2004). This mechanism could have impacted Lake Bysjön where past events of species introduction have been recorded (Eklöv & Eklövs Fiske & Fiskevård, 2003; Persson & Svensson, 2004). However, the absence of fish in the investigated pond (Sydvatten communication) invalidates the hypothesis of fish‐mediated transmission between the two locations. Alternatively, the use of working materials (e.g., engines, boots, and sands) can contribute to the dispersal of ephippia between water systems (Duffy et al., 2000; Hairston et al., 1995; Perrigo et al., 2012). However, under such dispersal we would have expected a higher admixed picture than the one observed in the present study.

Ricklefs (1987) proposes that regional processes (speciation and dispersal) have a greater effect on unsaturated communities than local processes (competition, predation, adaptation). Local sorting can strongly affect the spatial distribution of zooplankters (Hessen et al., 2019) governed by widespread endemism, provincialism and allopatric speciation (Hebert & Wilson, 1994), and founder effects (De Meester et al., 2002) within restricted areas. Newly arrived eggs are affected by drift during colonization, local inbreeding, and local genetic selection (Haag et al., 2006). Moreover, each system possesses seed‐banks of eggs that play a "rescue effect" (Gotelli, 1991) against local extinction and leads to competition with new incomers. Altogether, local sorting often leads to a reduce level of gene flow between populations. The moderate gene flow retrieved in the present study would suggest that local sorting may have an impact on the two neighboring populations.

Among local processes, we investigated the behavioral response of *D. magna* individuals among populations to solar radiation (UVR threat). Solar radiation is a strong selection pressure that constrains diel vertical migration in *Daphnia* species. Their natural avoidance of UV radiation is a protective mechanism against the acquisition of photodamages (Williamson et al., 2001). Their behavioral response consists in the increase of their swimming speed and distancing from the UVR source (Ekvall et al., 2015). Recent studies have shown that the swimming speed and behavior under UVR threat are inherited in *D. magna* across generations (Sha et al., 2020, this study). Moreover, the present study highlights a significant difference in swimming speed between populations under threat. The reason for the difference in swimming speed is beyond the scope of this paper. But, the high variance (Figure 5) and consistent individual response (Appendix S7) observed in swimming speed within each population indicate that different clones may respond differently to UVR threat.

Under the selection pressure inflicted by UVR on the swimming behavior of *D. magna,* populations follow the neutral phenotypic theory ((Fisher, 1958) in (Spitze, 1993)). Low variability regarding refuge demand was observed among populations, suggesting that this life history traits might evolve more slowly than the trait for swimming speed (different between populations under threat).

The presence of fish predators has an impact on the distribution of *Daphnia* individuals through the water column (Hansson & Hylander, 2009b). Moreover, the co‐occurrence of fish predation cues and UVR significantly affect the swimming speed in *D. magna* (Ekvall et al., 2020). Thus, in the two investigated water systems with different fish status (Lake Bysjön: fish predators; Pond: absence of fish), we expected a significant difference in swimming speed in *D. magna* between locations, characterized by a lesser response to light in the Lake Bysjön and POP1. Results confirmed the expectation by showing a significantly higher mean swimming speed in POP2.

A recent study demonstrated that *D*. *magna* can adapt rapidly (morphology, life history, swimming behavior) to different threats (predation, UVR) (Sha et al., 2020). We analyzed the adaptive genotypic diversity in *D. magna* using two markers associated with fish predation and general landscape stressors (i.e., fish predation, parasite infection, and land use)(Orsini et al., 2012). Our results showed that the adaptive genotypes were inherited within strain. However, deviations from this pattern included mutations principally located on the adaptive marker associated with fish predation. Moreover, high genotypic diversity over the fish predation marker occurred in particular in POP2 and over the general marker in POP1. Thus, the results indicate that genetic populations undergo different selection pressures in the two aquatic systems. The adaptive or evolutionary potential of populations is beyond the scope of this paper. But the significant difference in refuge demand in certain genotypes of *Daphnia* (Ba, Bd), principally associated with POP2 and the Pond, indicates strong selection for fish predation in POP1 and Lake Bysjön. Results are consistent with inferred swimming behavior and with the environmental history in Lake Bysjön (recreational location for fishing) and the hand‐made pond (exempt of fish but affected by anthropogenic activities).

Sha et al. (2020) further demonstrated that *D*. *magna* adaption to different threats (predation, UVR) occurred within three generations. Lineage analysis showed that 75% of the neutral multilocus genotypes and 71% of the adaptive genotypes were inherited within strains down to four generations. Deviation from expectation was associated with the occurrence of mutations and possibly with null alleles. Null alleles are the result of consistent amplification failure in PCR over a particular locus and can introduce substantial assessment errors (Dakin & Avise, 2004). In strains B41 and B42 for instance, mothers had the same neutral genotypes than their descendants but were assigned to Hybrid instead of POP1 (Appendix S5). We maintained these individuals as Hybrids but discarded them from the population genetic analyses, thus not affecting the connectivity estimates. We also maintained the two individuals in strain P31 for which a transition POP1‐POP2 was estimated, as the removal of the strain did not affect the genetic estimates. Globally, the observed mutations fall under microsatellite mutation pattern (i.e., frameshift, insertion/deletion, and principally step mutations) (Hancock, 1999; Selkoe & Toonen, 2006) with a net gain of heterozygosity as expected in asexual lines (Seyfert et al., 2008). Elevated microsatellite mutation rate would explain the punctual mutation events occurring in 13 out of 37 investigated strains. However, no clear mutation pattern was identified over the six inferred mutated lineages. Unexpectedly were the incongruent multilocus mutation patterns observed in both types of markers, at the origin of genotype and population cluster transitions (Appendix S5). Results call for genome‐wise complement analyses and longer time series to further understand the effect of different environmental pressures (e.g., predation, UVR, and predation and UVR) on the swimming behavior of *Daphnia* and other relative quantitative traits (e.g., fitness, organismal size), using an exhaustive set of adaptive markers (Orsini et al., 2012) coupled with genome‐wise (Bubac et al., 2020) and common garden experimentations.

In conclusions, our study unveils the existence of neighboring discrete populations connected by a moderate gene flow independent of water connectivity, highlighting the importance of wind‐ and animal‐mediated dispersal. The populations were under different selection pressures that reflects in the genotypic fingerprint of the few adaptive markers investigated. Moreover, these populations harbored diverse inheritable individual personalities associated with swimming behavior. Furthermore, our results suggest that despite strong local sorting, regional processes have an impact on isolated favorable habitats at local scales.

## CONFLICT OF INTEREST

The authors declare no conflict of interest.

## AUTHOR CONTRIBUTION

**Sylvie V. M. Tesson:** Conceptualization (lead); Formal analysis (lead); Funding acquisition (lead); Writing‐original draft (lead). **Yongcui Sha:** Conceptualization (supporting); Formal analysis (supporting).

## Supporting information

Appendix S1

Appendix S2

Appendix S3

Appendix S4

Appendix S5

Appendix S6

Appendix S7

## Data Availability

Data on sampling locations, behavior, microsatellite genotypes, and population diversity are provided as Appendices S1–S7.
