# Supplementary figures and images for: Population connectivity, dispersal, and swimming behavior in Daphnia
Source: Ecol Evol. 2021 Feb 28;11(6):2873–85. doi: 10.1002/ece3.7246 (PMC7981204; doi:10.1002/ece3.7246)

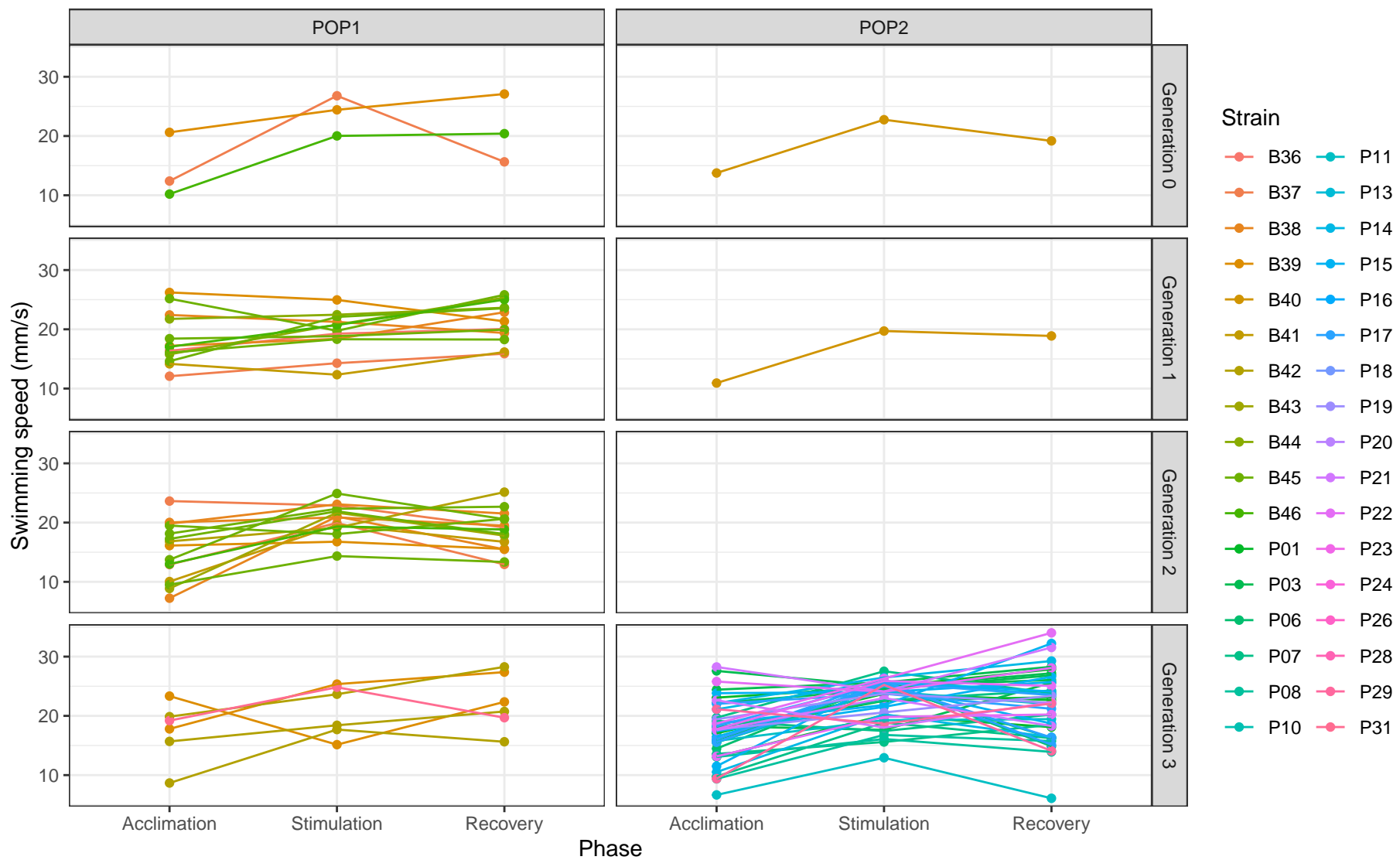

Supplement: Supplementary file 7 — Appendix S7 [file ECE3-11-2873-s007.pdf]
